# Spenectomy with Report of a Case

**Published:** 1913-08

**Authors:** E. C. Davis

**Affiliations:** Atlanta, Ga.


### Journal-Record of Medicine

Successor to Atlanta Medical and Surgical Journal, Established 1855
and Southern Medical Record, Established 1870

**OWNED BY THE ATLANTA MEDICAL JOURNAL COMPANY**

PUBLISHED MONTHLY

*Official Organ Fulton County Medical Society, State Examining Board, Presbyterian Hospital, Atlanta, Birmingham and Atlantic Railroad Surgeons' Association, Chattahoochee Valley Medical and Surgical Association, Etc.*

EDGAR BALLENGER, M. D., Editor
BERNARD WOLFF, M. D., Supervising Editor
A. W. STIRLING, M. D., C. M., D. P. H.; J. S. HURT, B. Ph., M.D.
GEO. M. NILES, M. D., W. J. LOVE, M. D., (Ala.); Associate Editors
E. W. ALLEN, Business Manager

**COLLABORATORS**

DR. W. F. WESTMORLAND, General Surgery
F. W. McRAE, M. D., Abdominal Surgery
H. F. HARRIS, M. D., Pathology and Bacteriology
E. B. BLOCK, M. D., Diseases of the Nervous System
MICHAEL HOKE, M. D., Orthopedic Surgery
CYRUS W. STRICKLER, M. D., Legal Medicine and Medical Legislation
E. C. DAVIS, A. B., M. D., Obstetrics
E. G. JONES, A. B., M. D., Gynecology
R. T. DORSEY, Jr., B. S., M. D., Medicine
L. M. GAINES, A. B., M. D., Internal Medicine
GEO. C. MIZELL, M. D., Diseases of the Stomach and Intestines
L. B. CLARKE, M. D., Pediatrics
EDGAR PAULIN, M. D., Opsonic Medicine
THEODORE TOEPEL, M. D., Mechano Therapy
R. R. DALY, M. D., Medical Society
A. W. STIRLING, M. D., Etc., Diseases of the Eye, Ear, Nose and Throat
BERNARD WOLFF, M. D., Diseases of the Skin
E. G. BALLENGER, M. D., Diseases of the Genito-Urinary Organs

VOL. LX. ATLANTA, GA., AUGUST, 1913. No. 5

### SPENECTOMY WITH REPORT OF A CASE.

BY E. C. DAVIS, M. D., ATLANTA, GA.

The function of the Spleen has been the bete noir of the profession and except for a few developments in the structural arrangement and its connection with blood cell repair and filtration, it still remains a much mooted subject.

One fact stands out undoubted, namely, that the Spleen is not essential to life, and whatever its function may be this can be performed by bone marrow or some other organ or organs when the Spleen has been removed.

Meta-physicians have ascribed temperamental disagreeableness to splenic disturbances and when a person was constantly disagreeable, they were spoken of as having SOME spleen.

In many of the toxic conditions the Spleen seems to act as a filter and in so doing frequently becomes much enlarged. This is especially true in some forms of malaria.

In leukocythemia we all know how much enlarged the Spleen becomes and in addition how promptly these patients succumb if any effort is made to remove the Spleen.

In certain conditions in which we have the so-called Wandering Spleen, this becomes enlarged and may cause symptoms so severe as to necessitate its removal. Again the Spleen like other organs is subject to malignant changes.

The case seen by me was one of Wandering Spleen which I confess I mistook for Fibroid, as it was found almost against the uterus, was of about the consistency of a Myoma and about twice the size of an ordinary cocoanut. The patient refused to come to Atlanta, so that we had to content ourselves with the physical examination without the laboratory findings.

A free incision was made, finding this mass to be the greatly hypertrophied Spleen; I then examined the liver to see if it was enlarged and passed my hand down to palpate the mesentery finding. No enlargement of the liver and no enlarged mesenteric glands, we lifted the Spleen out of the incision, ligated the Splenic artery and veins and removed it. We evidently allowed one of the veins to be overlooked for whenever we replaced the stump, it bled freely. This was again drawn out, caught and ligated. The patient was put to bed much shocked and for twenty-four hours had quite a squally combat. After this she began to improve and I append the report furnished by Dr. N. E. Benson, her family physician, which he has kindly furnished.

"I have only known her about two years. Her history is as follows, viz.: She began menstruating at 15 years, flow always very profuse. At one or more times she flowed enough to alarm her parents, who had her treated but with little success. Her bowels have, ever since she can remember, been "loose," three or four stools each day, much worse at monthly periods. Married at about nineteen. After birth of first child, about eight years ago, began to have female troubles which she said her doctors thought due to laceration. A sero-mucous discharge set up which lasted for some three years, she having conceived a second time. At about this time, about five years, she noticed this lump in her side which was barely perceptible. After this second child was born it could be felt very plainly. Her physician then told her it was the spleen and it would never amount to anything, but she still had the heavy weight in the side and at times pain. About two years ago, she was treated by some traveling quack, not getting any relief. After this time I first saw her. She was very much emaciated, complaining of a dull ache in left side in region of ovary. On examination the spleen could be made out some two or three inches below border of ribs. She had a very offensive discharge from vagina. Some albumen in water and mucus in most every action from bowels, the latter always being much worse during menstruation. This condition improved some, but never entirely well. In summer of 1912 she went to seacoast for three weeks and while there had an attack of malarial fever. Came home about first of August, was in bed two weeks. This enlargement had increased now and almost gotten lower down until I began to think of fibroid, however, after two weeks of chills every three or four days, she was dismissed. In one week, the last week of August, I was called again. She had high fever. Had had another chill. On examination, found the "lump" as large as the largest sized cocoanut. She was given anti-malarial treatment of all kinds, but was in bed two or three weeks until you saw her and operated. After operation her circulation was disturbed quite a bit. Pulse rate for three weeks anywhere from 110 to 148. She was carried home in about two weeks after operation. Has not been sick since. Her bowels have become regular, moving once a day. But here will say that it is necessary for her to take pepson all the time. I can't account for it, but if left off she invariably has pain in bowels and sides and also becomes loose and some mucus. The same medicine had no effect before she was operated on. The discharge from the vagina is very much less. The kidneys absolutely normal, circulation 88, respiration 22. Says she feels as well as anyone. Weighs 121 lbs., former weight being 98 to 104. She is not very much more irritable than before."

